# VisiEAU 2018—A Vision for Water in Haiti, 2018^1^

**DOI:** 10.3201/eid2410.180693

**Published:** 2018-10

**Authors:** Jocelyn M. Widmer, Florence Sergile, Yvens Cheremond, J. Glenn Morris

**Affiliations:** University of Florida, Gainesville, Florida USA (J.M. Widmer, F. Sergile, J.G, Morris, Jr.); Université d’Etat d’Haiti, Port-au-Prince, Haiti (Y. Cheremond)

**Keywords:** water, water management, water safety, conference, VisiEAU 2018, public health, sanitation, hygiene, waterborne diseases, bacteria, Haiti

Water is at the core of human existence, and availability and quality serve as key drivers for public health, agriculture, and development. As noted in the keynote address of the VisiEAU 2018—A Vision for Water in Haiti, 2018, conference by Professor Wilson Celestin of the Université d’Etat d’Haiti, challenges with water in Haiti span a range of issues: a lack of coordination among governmental and private institutions at the national and local level; infrastructural limitations for storage, treatment, and distribution systems; data deficiencies to support decision making; disjointed legislation dealing with rights to water; fragmented finance mechanisms for infrastructure enhancement; and education barriers at the community and household level that lead to pervasive health threats.

The issue with water in Haiti is not that the resource is insufficient, but that it is inadequately distributed and poorly managed. Water quality has been degraded because of social and demographic shifts over time, in conjunction with acute and chronic impacts from land use, infrastructure deterioration, and changes in climate. Sixty-two percent of urban and 34% of rural residents have access to distributed water (water collected/distributed to persons living within a radius of 500 m around a water point). However, during 2000–2012, a smaller percentage of the population in both settings used an improved water source (defined as a source protected from exterior contamination). Water treatment practices are not yet widespread, and nearly 3 in 10 households drink untreated water (*1*). Saline contamination threatens major urban aquifers such as the Plaine du Cul-de-Sac Aquifer, which provides water to 60% of the population living in the Port-au-Prince metropolitan area, because of more than a century of pumping practices that are out-pacing the ability of the aquifer to recharge.

The population of Haiti is currently >10 million and expected to increase to 14 million during the next 20 years. Consumption rates and quality of water resources available to meet growing demand place an increasing emphasis on the need for water governance and management. The Framework Law of 2009 (*1*) gave rise to Direction Nationale de l’Eau Potable et de l’Assainissement under the Ministry of Public Works for dealing with regulation of potable water through a decentralized system of regional authorities. However, other aspects of water management fall under the jurisdiction of 6 national ministries, each governed by its own rules and functioning within its own budget, making it difficult to develop a coordinated approach. This situation is further complicated by the >50 nongovernmental organizations (NGOs) actively participating in the water and sanitation sector.

Although targets for water and sanitation have been included in Sustainable Development Goal 6, the challenge lies in identifying effective interventions that respond to the diversity of water delivery, which includes wells, community treatment centers, kiosks, water trucks, water sachets, and bottled water products. The presumption that groundwater is potable and does not necessitate further treatment when derived from an engineered source is not necessarily true, a fact that underscores the need for secondary treatment approaches at the community level that balance quantity, quality, and accessibility with cost. Diarrheal diseases remain a major public health challenge; illness are often caused by pathogens transmitted by contaminated water.

## Key Objectives of the Conference

VisiEAu 2018 sought to define natural resource characteristics specific to water in Haiti; explore water governance, rights, and legislation in Haiti and across Hispaniola; highlight systems of water management, infrastructure, and distribution; share examples of research activities that investigate transmission of waterborne pathogens and the effect of interventions to date; and identify opportunities to integrate technology into water management for improved health outcomes. The conference also aimed to forge strategic partnerships among relevant government ministries, NGOs, and university researchers to transition policy dialogue into action; and then for this action to inform a new era of best practices in teaching through the creation of a new Water Sanitation and Hygiene Institute at the Université d’Etat d’Haiti, which will have implications for water, sanitation, and hygiene throughout communities across Haiti.

## Agenda and Participants

The first day of the conference focused on the unique geographic, hydrologic, social, cultural, and political context in which water resources are managed and used. The second day of the workshop focused on the nexus between water and health, honing in on the environmental effects on public health related to waterborne diseases. A list of speakers and their topics is provided (Table). We would note, in particular, the participation of representatives from the Haitian Ministries of Environment; Commerce and Industries; Agriculture, Natural Resources and Rural Development; Public Health and Population; and Public Works; the Rector’s Office and Colleges of Medicine and Pharmacy, Engineering, and Agriculture at the Université d’Etat d’Haiti; the Emerging Pathogens Institute, Water Institute, and School of Natural Resources and Environment of the University of Florida; the University of Quisqueya; the US Centers for Disease Control and US Embassy; NGOs, including Water Missions; and private companies, including Northwater International, Charles Fequiere SA, and beverage companies.

**Table:** Conference speakers and topics at VisiEAU 2018—a vision for Water in Haiti, 2018*

Session and speaker	Topic	Affiliation
First day keynote address		
Wilson Celestin	Integrated water management: an absolute need for Haiti	Université d’Etat d’Haiti
Water natural resource: volume, inventory, and mapping		
Bobby Emmanuel Piard	Mapping water	Centre National de l’Information Géo-Spatiale
Pierre Adam	Recharge of the water table	Université d’Etat d’Haiti
Lionel Rabel	Water resources in Haiti	Université d’Etat d’Haiti
Tom Frazer	Water, ecology, and environment	University of Florida
Wendy Graham and Niangona Gonomy	Workshop A. Water as a natural resource workshop	University of Florida and Université d’Etat d’Haiti
Who owns water? Governance, rights, and legislation		
Jean-André Victor	Water war between Haiti and the Dominican Republic	Université d’Etat d’Haïti and Association Haïtienne de Droit de l’Environnement
Marie Jane Angelo	Water law and management: perspective from the United States	University of Florida
Astrel Joseph	Current state of water governance in Haiti	Ministry of Environment
Jean-Marie Raymond Noël	Access, social and communal water management	Université d’Etat d’Haiti
Montes Charles and Samantha Jacob	Workshop B. Who owns water? Governance, rights, and legislation	Université d’Etat d’Haiti and Ministry of Agriculture; University of Florida
Second day keynote address		
Evans Emmanuel	Environmental impacts on public health: medical geology contribution to understanding chemical risks associated with water	Quisqueya University
Water management, structures, and distribution		
Cinthia J.-B. Blaise	Water quality, human and environmental health in Greater Port-au-Prince	Direction Nationale de l’Eau Potable et de l’Assainissement
James K. Adamson	A recent evaluation of the Plaine du Cul-de-Sac Aquifer, and its potential to serve Canaan	Northwater International
Johnny François	Developing a community drinking water system: Perspective of a nongovernmental organization	Water Mission
Alexandre Laraque	Water value chain and business	Caribbean Bottling Company
One Health		
Richard Gelting	WASH and waterborne diseases in Haiti	US Centers for Disease Control and Prevention
Jacques Blaise	Zoonotic parasites transmission risks in drinking water in Haiti	Université d’Etat d’Haiti
Eric Nelson and Molly Klaman	How might we best train first responders to waterborne infectious disease outbreaks?	University of Florida
J. Glenn Morris	Research on transmission of waterborne pathogens in Haiti	University of Florida
Jean Allain Darius and Réginald Clvéus	Workshop C. WASH and water management	US Centers for Disease Control and Prevention and UNICEF
Elda Déronette and Eric Nelson	Workshop D. Technology and water security: how might we...	Université d’Etat d’Haiti and University of Florida
Stace Maples	How might we better rapidly map waterborne infectious disease outbreaks?	Geospatial Manager at the Stanford University Geospatial Center
Joshua Wyrtzen	How might poor communities be empowered to report water infrastructure problems?	SeeClickFix

*UNICEF, United Nations Children’s Fund; WASH, water, sanitation, and hygiene.

The diversity of perspectives represented among participants encouraged rethinking of the matrix of responsible water use and management. Ministry representatives outlined strategies to prioritize and integrate the issue of water into the national mandate and to take the lead in coordinating diverse groups of actors involved in water use. Environmental and public health scientists described the exchange between hydraulic, geologic, and urban characteristics of water that affect human health. Data engineers from the government and academia showcased prototypes that are making water-related geospatial data available to decision makers in policy and clinical settings and empowering community residents. NGOs told stories in which communities were sustainably engaged in planning, inventorying, and monitoring water systems through transparent processes meant to improve water access in various geographic areas across the country.

## Key Messaging as a Way Forward with Water Management

The conference produced several key messages around a call to action for improved water management. Chief among these messages was that integrated water management is essential to understanding water, which includes access, quality, and pricing, and better coordination among the 6 ministries, 50 NGOs, and others working in the water sector in Haiti. Information is needed to improve decision-making processes, which can only occur through improved mechanisms for data sharing alongside innovative uses of emerging field-based technologies. Water, sanitation, and hygiene and water management are critical to overcoming the enormous burden of disease attributed to waterborne pathogens. Innovations are occurring in system design, technology deployment for monitoring and evaluation, and capacity building. However, there remains an educational imperative to train a new generation of professionals to work in water management and also train consumers in rural and urban communities across the country. This imperative is reflected in efforts by Université d’Etat d’Haïti to enhance the Water and Sanitation Institute and develop interdisciplinary programs to foster research and education. The opportunity for partnerships to be strengthened among policymakers, researchers, program implementers, and educators is central to achieving these key goals.

## References

[1] Cayemittes M, Busangu MF, de Dieu Bizimana J, Barrère B, Sévère B, Cayemittes V, et al. 2013. Mortality, morbidity and utilization survey, Haiti, 2012 [in French]. Calverton (MD): MSPP, IHE, and ICF International [cited 2018 Jul 19]. https://dhsprogram.com/pubs/pdf/FR273/FR273.pdf

